# A Review of Transnational Migrant Entrepreneurship: Perspectives on Unequal Spatialities

**DOI:** 10.1515/zfw-2021-0004

**Published:** 2022-10-06

**Authors:** Laure Sandoz, Christina Mittmasser, Yvonne Riaño, Etienne Piguet

**Affiliations:** University of Neuchâtel Geography Institute nccr – on the move Espace Tilo-Frey 1 2000 Neuchâtel Switzerland; University of Neuchâtel Faculty of Letters and Humanities Institute of Geography Espace Tilo-Frey 1 2000 Neuchatel Switzerland; University of Neuchâtel Faculty of Letters and Humanities Neuchatel Switzerland; University of Neuchâtel Faculty of Letters and Humanities Neuchatel Switzerland

**Keywords:** Transnationalism, Entrepreneurship, Migration, Mobility, Spatiality, Inequality

## Abstract

The spatialities of migrant entrepreneurship have changed dynamically in recent decades. Movements and exchanges transcend national borders more than ever, and transnational migrant entrepreneurship has become a burgeoning field of research. Yet, knowledge is dispersed across disciplines, and an understanding of contemporary spatialities is limited. We review 155 articles published in English, French, German, and Spanish since 2009, thereby providing an overview of existing knowledge on transnational migrant entrepreneurship and suggesting avenues for future research. We identify five current topical areas of research: (1) the business advantages of transnational migrant entrepreneurship, (2) the determinants of becoming a transnational migrant entrepreneur, (3) the transnational networks of migrants, (4) the economic impacts of transnational migrant entrepreneurship on home and host countries, and (5) whether local environments enable or deter entrepreneurial success. Building on our synthesis of the most recent literature, we propose three crucial dimensions which have been under-researched in past and current work, and which address the diversity of geographical locations, spatial connections, and spatial mobilities involved in transnational migrant entrepreneurship. Moreover, we put forward a set of questions for future research which will advance a comprehension of unequal opportunities among transnational migrant entrepreneurs.

## 1 Introduction

Migrant entrepreneurship is of increasing scientific and policy interest today. Rates of business creation and self-employment in migrant communities appear to be higher than the national averages in many countries and play an important role in local economies (IOM, 2019; Juchno & Agafitei, 2017; UNCTAD, 2018). Furthermore, the spatialities of migrant entrepreneurship have changed dynamically in recent decades. Transnational life course trajectories, coupled with contemporary social and technological transformations, have created new opportunities for migrants to move goods, capital, and ideas across national borders for business purposes (Ambrosini, 2012; Portes et al., 2002). Transnational migrant entrepreneurship therefore addresses economic geography’s central concern, which is to study the relationship between economic activities and the spaces where they are carried out and circulate (Barnes, 2009).

In 2009, Yeung stated that transnational entrepreneurs should not be viewed “as merely localised agents of economic change, for they embody different spatialities of economic action and processes” (2009, p. 211). He advocated for paying more attention to how cross-border entrepreneurship produces transnational spaces and connects multiple locations. Today, transnational migrant entrepreneurship is a burgeoning field of research that combines economic, business, and social science approaches (Harima & Baron, 2020; Muñoz-Castro et al., 2019; Zapata-Barrero & Rezaei, 2020). Yet, knowledge is dispersed across disciplines.

We will advance this field of research by critically discussing the literature on transnational migrant entrepreneurship based on a database of 155 scientific journal articles published since 2009, thereby providing an overview of existing knowledge and suggesting avenues for future research on this topic. While acknowledging that transnational approaches have significantly improved our understanding of migrant entrepreneurship, we recognise that a *spatialities perspective*—concerned with how this phenomenon is positioned in space—needs further development. This is important because entrepreneurs do not act, exchange, and move in a vacuum but in concrete material spaces. We contribute to this endeavour by identifying three under-researched dimensions for future research which address the diversity of *geographical* *locations*,* spatial connections*, and *spatial mobilities* involved in transnational migrant entrepreneurship. Moreover, we emphasise that *unequal opportunities* available to transnational migrant entrepreneurs depend on social markers such as class, nationality, gender, and ethnicity.

The article comprises six parts. Following the introduction, the second section briefly introduces our understanding of transnational migrant entrepreneurship in relation to spatiality, and the third describes our methodological approach. The fourth part identifies major topical areas of study in the literature: (1) the business advantages of transnational migrant entrepreneurship, (2) the determinants of becoming a transnational migrant entrepreneur, (3) the transnational networks of migrants, (4) the economic impacts of transnational migrant entrepreneurship on home and host countries, and (5) whether local environments enable or deter entrepreneurial success. The fifth section discusses future avenues of research, and further develops our proposed dimensions to examine contemporary spatialities of migrant entrepreneurship. The conclusions set the findings in a wider context.

## 2 Defining Transnational Migrant Entrepreneurship in Relation to Spatiality

The term “transnational entrepreneur” was first popularised by Portes et al. (2002), who applied it to “self-employed immigrants whose business activities require frequent travel abroad and who depend for the success of their firms on their contacts and associates in another country, primarily their country of origin” (p. 287). Studies on this specific form of entrepreneurship lie at the crossroads between sociologically oriented research on ethnic economies and economic/business-oriented research on international entrepreneurship. In this article, we use the term transnational migrant entrepreneurship for the following reasons. Compared with international entrepreneurs, transnational migrant entrepreneurs have migration experience, which has been recently recognised as key asset for their business activities (Elo et al., 2018). Moreover, international entrepreneurship is predominantly concerned with firm analysis whereas transnational entrepreneurship examines the cross-border activities of individual entrepreneurs (Drori et al., 2009; Etemad, 2018). Transnational migrant entrepreneurs can also be distinguished from ethnic entrepreneurs based on their ability to simultaneously engage with multiple places and develop ties that extend beyond a single ethnic economy (Henn, 2012; Honig, 2020). In current studies, however, the categories of transnational, international, and ethnic entrepreneur often overlap. These different research traditions have only recently begun to communicate with one another, and the interdisciplinary field of transnational migrant entrepreneurship is starting to become more unified (Harima & Baron, 2020). Following the most recent developments, we define transnational migrant entrepreneurs as former and current migrants who use transnational migration experiences and networks to develop businesses that are active in more than one country (c.f. Sinkovics & Reuber, 2021). Empirically, this definition can be applied using both quantitative (e. g. surveys) and qualitative (e. g. interviews and focus groups) methods.

The multiplicity of places, connections, and mobilities within which transnational business activities occur lead us to rethink the notion of spatiality. In transnational settings the spatial ontology needs to shift “from a static and circumscribed characterization of migrant economies to a more dynamic and flexible one” (Valenzuela-Garcia et al., 2018, p. 49). How can we then conceptualise space and spatiality in this context? Concepts of space are at the heart of geography research. Although abstract and difficult to grasp, “it is precisely the multiplicities and heterogeneous nature of space and spatiality—as abstract and concrete, produced and producing, imagined and materialised, structured and lived, relational, relative and absolute—which lends the concept a powerful functionality that appeals to many geographers” (Shoorcheh, 2019, p. 64). Broadly speaking, we understand spatiality as an entanglement of materiality, which refers to the physical characteristics of places as well as social practice and symbolic meaning. Therefore, the everyday actions and experiences of individuals transform material spaces into social spaces (Riaño, 2017).

In the context of transnational migrant entrepreneurship, specific spatialities arise when individuals simultaneously act in multiple *locations* and progressively develop meaningful social, economic, political, and symbolic *connections *by conducting and maintaining diverse* mobilities* across borders. In this article we attempt to demonstrate how a focus on these spatialities can advance our understanding of transnational migrant entrepreneurship. Thus, we propose to adopt a processual and relational understanding of space, with a focus on the social interactions that (re)produce space over time, as well as the power hierarchies that shape spatial settings and individuals’ options (Massey, 2005). As we will show through our literature review, this conceptual approach contributes to the field of transnational migrant entrepreneurship by highlighting the role of unequal spatialities in shaping different opportunities and constraints among transnational migrant entrepreneurs.

## 3 Methodology

We created and reviewed an interdisciplinary corpus of 155 journal articles on transnational migrant entrepreneurship published since Yeung’s work in 2009, applying rigorous methods and best practices to identify the most relevant literature (Haddaway et al., 2020). Focusing exclusively on journal articles is a pragmatic choice frequently practised in literature reviews (Aliaga-Isla & Rialp, 2013). We combined “automated” methods (using alerts and information-gathering software such as Feed Informer to both monitor existing literature and discover new publications) and “manual” methods (adding references from ongoing keyword searches in online databases and from the bibliographies of already-obtained articles). A complete list of databases and journals monitored via automated research is provided in the online appendix to this article. Our corpus includes scientific journal articles in English, French, German, and Spanish (the main languages spoken by our team members).

We selected journal articles on transnational migrant entrepreneurship based on the definition presented in the previous section. To begin with, we researched academic databases and journals using the keywords “transnational”, “migrant”, “entrepreneurship”, and synonyms of these (e. g. “transnationalism”, “migration”, “immigrant”, “entrepreneur”, “business”, “start-up”, “firm”) in English, French, German, and Spanish (see online appendix). We then checked each article to ensure that they actually covered on the topic, which led us to exclude several articles because the transnational dimension of migrant entrepreneurship was not explicitly mentioned. In a third step, we manually reviewed the bibliographies of the selected articles to identify references that the automatic search had missed. We repeated this process with newly selected articles until we could find no further salient references. These steps led us to identify 155 relevant articles (listed in the online appendix) published between 2009 and 2019, and originating from various disciplines (mainly economics, business and management, sociology and geography). Despite the inclusion of non-anglophone articles, only seven are in French, German, or Spanish, thus highlighting the predominance of English-language publications in the field. Nevertheless, we believe that this corpus allows us to develop an extensive overview of the state of the art. In addition to our main corpus, we also considered a special issue of the *Journal of Entrepreneurship and Innovation in Emerging Economies* on transnational migrant entrepreneurship (2020) to include the most recent literature.

We reviewed each publication to identify key definitions of transnational migrant entrepreneurship, research questions, theoretical lenses, regional contexts, methods, results, and research gaps. Our team collaboratively discussed and synthesised this information in an iterative process. We first developed an overview of our knowledge about transnational migrant entrepreneurship. We then applied a content analysis methodology (Mayring, 2014) to inductively develop a set of preliminary codes for each article (examples of codes include: “choice of location for transnational activities”, “role of governments in promoting transnational entrepreneurship”, “commitment to the home country through economic actions”, etc.), which we subsequently reorganised into broader categories. Inspired by other literature reviews (Piguet et al., 2018), we worked with specific categories to ensure coherent analysis. This process enabled us to identify five main topical areas of research on transnational migrant entrepreneurship, which will be discussed in the next section.

## 4 Major Topical Areas of Research in Recent Literature on Transnational Migrant Entrepreneurship

This section presents an overview of five major topical areas of research—including results and gaps—on transnational migrant entrepreneurship since 2009, highlighting the strengths and limitations of current research: the first focuses on identifying the specific advantages of transnational migrant entrepreneurship and aims to empirically test the extent to which migration experiences can, under certain circumstances, constitute an asset for transnational business-related activities; the second attempts to understand the reasons, determinants, and motivations that lead migrants to engage in transnational businesses; the third analyses the composition of migrants’ personal networks and their potential utility in transnational business-related activities; the fourth assesses the economic impact of transnational entrepreneurs on the places where they conduct business; and finally, the fifth examines the role of local environments in supporting or deterring transnational migrant entrepreneurship.

### 4.1 Specific Advantages of Transnational Migrant Entrepreneurship

Inspired by Schumpeter (1974), research on entrepreneurship often distinguishes between necessity-driven and opportunity-driven entrepreneurs, arguing that the latter drive innovation and reforms (Baltar & Icart, 2013; Zapata-Barrero & Rezaei, 2020). While initial research on migrant entrepreneurship focused on “ethnic minorities” as being more likely to become self-employed out of necessity due to exclusion from the labour markets of host countries, recent studies have criticised this deficit-based approach (Elo et al., 2018; Garrido & Checa, 2009). They show that the ethnic and exclusion lenses do not sufficiently capture the complex realities of today’s transnational migrant entrepreneurship because they fail to recognise migrants’ abilities to capitalise on opportunities beyond ethnic and national boundaries (Drori et al., 2009; Muñoz-Castro et al., 2019). Scholars in business and management studies have developed a positive view of transnational entrepreneurship, considering migration experiences advantageous to expanded business opportunities and calling for a “reconceptualisation of migrants from an economic burden to a source of economic activity and export earnings” (Baklanov et al., 2014, p. 68).

The positive conceptualisation of transnational migrant entrepreneurship builds on the notion of “dual embeddedness”, which posits that migrant entrepreneurs have a competitive advantage (Elo & Vincze, 2019; Emontspool & Servais, 2017) due to their ability to access “economic resources, education and social networks, and exposure to social lifestyles” in the home and host countries (Dahles, 2013, p. 386) For some authors this unique position drives innovation (Harima, 2014), success (Ojo, 2012), development (Ashourizadeh et al., 2016), emancipation (Villares-Varela & Essers, 2019), prestige (Ottati, 2014), and socioeconomic integration (Lin & Tao, 2012).

While the literature often implies that dual embeddedness automatically leads to economic success and other promising outcomes, we need to remain sceptical as long as this process is not explained. In practice, authors who stress the added economic value of transnational entrepreneurs tend to select case studies involving highly skilled people in the technology, consulting, and educational sectors (Brzozowski et al., 2014; Crick & Chaudhry, 2013). By contrast, authors with a more critical approach focus on less privileged migrants working in low-profit sectors. For example, Eckstein and Nguyen (2011) analysed Vietnamese manicurists in the US who created a transnational market for professional nail care without significantly improving their long-term earning potential. Similarly, Munkejord (2017) showed that migrant women in rural Norway who build diverse businesses are motivated mainly by developing their sense of regional belonging and fulfilling personal aspirations rather than improving their economic situation. This raises the question of whether or not dual embeddedness is the key to success for some transnational entrepreneurs, and under what circumstances migrants profit from it (Jones et al., 2010; Wahlbeck, 2018). In other words, this interrogates whether transnational connections are a main driver of social mobility and economic development or simply reflect existing privileges and inequalities among entrepreneurs. Furthermore, we agree with other scholars (Harima & Baron, 2020; Solano, 2020) that transnational entrepreneurship is not simply a relationship between the origin and destination countries. Contemporary migrants have the potential to simultaneously connect with various countries, given their multiple mobility experiences and global connections. In their study of Bukharian Jews in the diaspora, Elo and Dana (2019) speak of “multiple embeddedness” to describe the extended family, ethno-religious, cultural, and social ties that shape this specific transnational entrepreneurial community. Finally, some authors contest the simplistic view of transnational migrant entrepreneurship as only positive by emphasising the enduring hardships and inequalities that many entrepreneurs face when developing transnational economic activities (Åkesson, 2016; Wahlbeck, 2018). Thus, we suggest that future studies need a more differentiated understanding of the diverse transnational connections created, developed, and maintained by entrepreneurs from more and less privileged social backgrounds.

### 4.2 Motivations and Determinants of Transnational Migrant Entrepreneurship

Many authors explore the reasons and necessary conditions that lead some migrants to engage in transnational business activities (Portes & Martinez, 2020). Researchers in business and management studies tend to associate transnational migrant entrepreneurship with specific individual attitudes, skills and motivations such as the ability to identify business opportunities (Lundberg & Rehnfors, 2018), the desire for capital accumulation (Ribeiro et al., 2012), and the propensity to take risks (Urbano et al., 2011). Although criticised by some because of reified and static definitions of culture (Glick Schiller & Çağlar, 2013; Liu, 2012), several authors highlight that specific nationalities are particularly prone to engage in entrepreneurship (Dimitratos et al., 2016; Goktan & Flores, 2014). A distinction can be drawn between explanations linking a specific national culture with the emergence of entrepreneurship, and explanations that view cultural resources as a potential asset for business creation.

Social scientists tend to focus more on the determinants of social structure, unpacking the social position of transnational migrant entrepreneurship regarding variables such as education, gender, ethnicity, length of stay, and financial security (Nkrumah, 2018; Webster, 2017). Using Bourdieu’s capital approach, several authors highlight the importance of social, symbolic, economic, and cultural resources to business development (Åkesson, 2016; Nowicka, 2013), stressing the unequal distribution of resources between individuals and groups as well as the specific power relations embodied in social hierarchies. For example, in her study of Polish entrepreneurs in Germany, Nowicka (2013) developed a theoretical model to analyse how intersections and conversions of different forms of capital across national borders, such as the ability to mobilise specific resources and social networks transnationally, influence the social positions that migrants can achieve in both their home and host country. Other authors analyse enabling and disabling factors related to specific socioeconomic environments, and highlight the obstacles faced by specific categories of migrant entrepreneurs such as women or refugees (Halilovich & Efendić, 2019; Lee & Lee, 2020). For example, in a study examining the business start-up process of Chinese and Turkish restaurant owners in Finland, Katila and Wahlbeck (2012) show that the ability of migrant entrepreneurs to activate transnational networks is connected to specific migration trajectories and entry patterns. In another study of Latino entrepreneurs in the US, Poblete (2018) discusses the role of discrimination, highlighting that negative experiences in the host countries can strengthen ties with the country of origin and thus motivate migrants to engage in transnational business activities.

However, a comprehensive examination of the role of spatial mobility in transnational business creation is still lacking. Overall, scholars engage with different determinants of cross-border entrepreneurial activities but pay insufficient attention to the fact that migrants have unequal opportunities to travel and move goods, services, and ideas into transnational spaces. The processes of spatial mobility involved in transnational migrant entrepreneurship should not be taken for granted. Moreover, questions concerning the relationships between (im)mobility, inequality, and space are rarely raised. Examining the interplay between geographical location and social position would enable scholars to reveal the complex spatial nature of the mechanisms that shape transnational migrant entrepreneurship. This focus would cast new light on how entrepreneurs’ options are structured by concrete locations and their connections, and how spatially situated processes of mobility and immobility participate in the (re)production of global social inequalities.

### 4.3 Social Ties and Networks of Transnational Migrant Entrepreneurs

Authors in all disciplines have developed a growing interest in how transnational ties and networks impact the performance of migrant entrepreneurs, with particular interest in social contacts between migrants and their countries of origin (Elo & Hieta, 2017; Pruthi et al., 2018), diasporic networks (Evansluong et al., 2019; Fossati, 2019), and returnees’ social contacts in former host countries (Åkesson, 2016; Santamaria-Alvarez et al., 2018).

Following Chen and Tan’s (2009) influential contribution to this topic, some authors provide evidence that transnational networking significantly improves entrepreneurial performance (Kariv et al., 2009; Mustafa & Chen, 2010), and that this positive impact increases as ties strengthen (Patel & Terjesen, 2011). Yet, other authors obtain more ambiguous results by highlighting transnational migrants’ risk of disconnection from home country networks (Nawojczyk & Nowicka, 2018; Quan et al., 2019). Several scholars discuss the argument first developed by Portes et al. (2002) that local embeddedness enhances transnational connections because it provides a stable basis to support transnational economic engagements (Brzozowski et al., 2017; Sequeira et al., 2009).

This area of research points towards the interdependence of local and transnational forms of belonging and the relationship between local and transnational socioeconomic integration. We propose that a spatial perspective, which analyses how distant places become connected through the geographically situated activities of entrepreneurs, and emphasises the concrete locations, mobilities, and processes that bring these connections to life, would further advance our understanding of the role of networks in transnational migrant entrepreneurship. It would also enable a closer analysis of the power dynamics that shape the relationships between transnational migrant entrepreneurs and network members. Many studies highlight the importance of private and family networks to transnational entrepreneurs’ business development (Henn, 2012; Liu et al., 2020), thus pointing to the role of emotional connections and trust in business-making processes. Zani (2019), for example, speaks of “emotional space” to describe the physical and virtual transnational economies that Chinese migrant women develop using digital technologies. In doing so, women maintain ties between China and Taiwan and cope with economic discrimination. Similarly, the literature on diasporic entrepreneurial communities points to the important role of attachment and identification to the host country, as well as feelings of duty and obligation towards co-nationals and diaspora members, as central motivations to engage in both pecuniary and non-pecuniary investment (Osaghae & Cooney, 2020).

Although self-employment is often associated with independence, research shows that mutual dependencies exist between transnational entrepreneurs and network members, some of which are more beneficial than others (Fuller-Love & Akiode, 2020). Therefore, we call for a more diverse portrayal of the complex social and spatial dependencies involved in transnational migrant entrepreneurship, which carefully addresses individual experiences in both private and professional spheres.

### 4.4 Economic Impacts of Transnational Migrant Entrepreneurship on Host and Home Countries

Evaluating the impact of transnational businesses on the economic development of a region or country in terms of resource flows, knowledge transfers, foreign investments, and employment creation is of central interest. Most discussions on development are grounded in the “brain-drain/brain-gain” debates, which examine the positive and negative effects of migration flows from poor to rich countries (De Silva, 2015; Veréb & Ferreira, 2018).

Some authors support Saxenian’s (2007) idea of “brain circulation”, which describes the positive impacts of transnational entrepreneurial activities on the economies of both home and destination countries (Portes & Yiu, 2013; Terjesen & Elam, 2009). Riddle et al. (2010) study a business incubator for transnational entrepreneurs in the Netherlands, and highlight how migrants from Afghanistan, Ethiopia, Ghana, Morocco, Surinam, and Turkey receive support to develop cross-border businesses that generate employment and investments in both residence and origin countries. Other authors focus exclusively on the economic impact of transnational entrepreneurs on their home countries. Ojo et al. (2013), for example, analyse the inclination of Sub-Saharan African entrepreneurs in the UK to promote business development in their countries of origin through investment strategies, and observe positive consequences in terms of innovation and opportunity creation.

However, authors such as Santamaría-Alvarez and Śliwa (2016) obtain less optimistic results. Studying the entrepreneurial activities of Colombian emigrants to the US, they argue that if transnational networks are fragmented and governmental support is lacking, their transnational entrepreneurship activities have little impact upon their home country’s economy and society. Nkongolo-Bakenda and Chrysostome (2013) discuss the importance of altruistic motivations and the need for social recognition from the home country to explain why certain entrepreneurs engage in business activities in environments that other investors would consider too hostile and risky. In line with other authors (e. g. Brzozowski et al., 2014) they posit that socioeconomic development not only depends on the actions of individual entrepreneurs but on state policies and other contextual factors within the home and host countries. Several studies focus on how supportive diaspora policies and entrepreneurship programmes for migrants and returnees contribute to economic development (Rezaei & Goli, 2020; Zapata-Barrero & Hellgren, 2020), while others highlight the obstacles and institutional voids that prevent successful investments (De Silva, 2015).

In economic geography and related disciplines, many scholars address the question of how individual migrant entrepreneurs contribute to the development of business clusters and entrepreneurial ecosystems at a local level, asking to what extent and under which conditions they can generate positive dynamics of economic development (Maceda Rodriguez & Vazquez Vazquez, 2016). Schäfer and Henn (2018) propose a three-stage model in which highly skilled returnees become entrepreneurial pioneers. These returnees act as role models for local entrepreneurs and diaspora members, thus promoting subsequent business engagement and local economic growth. In addition, these authors argue that the role of network linkages and connections between entrepreneurial ecosystems situated in different locations needs to be further researched.

Overall, how spatial dependencies are created through the practices of transnational entrepreneurs across the globe and the impact of historical hierarchies between the places involved are topics rarely evoked. Paying closer attention to “the inequality of power and forms of economic domination that characterises the relations between rich and poor countries” (Calhoun, 2002) and shifting the focus from an economic growth perspective to a spatial inequalities perspective would advance our understanding of transnational entrepreneurship at both local and transnational levels.

### 4.5 The Role of Local Environments

Using economic or political science approaches, and inspired by Everett Lee’s theory of migration (1966), most authors distinguish between location-related push and pull factors that may influence a migrant’s engagement in transnational entrepreneurship (Honig, 2020). Pull factors include positive location-specific elements such as supportive policies, favourable market conditions, or personal networks, whereas push factors refer to obstacles and constraints such as economic crises, competitive pressures, or unemployment. This distinction highlights the fact that transnational entrepreneurship can be a way to balance negative conditions in one location with positive conditions in another. Brzozowski et al. (2014) show, for example, that the home country’s institutional and socio-economic characteristics (such as macroeconomic stability, level and quality of education, level of corruption, and level of entrepreneurial endowment) play a crucial role in shaping the performance of transnational immigrants’ business activities in the Italian information and communications technology (ICT) sector.

“Mixed embeddedness” is another major theoretical framework used to discuss the impact of locations on transnational migrant entrepreneurship. Proposed by Kloosterman et al. (2010), this approach combines the micro-level of an entrepreneur’s resources with meso-level opportunity structures and macro-institutional frameworks. Recent publications have tried to adapt this approach by analysing the interconnectedness of the various environments in which migrants operate (Solano, 2020). Building on interviews with Vietnamese business owners based in London, Bagwell (2018) studies transnational mixed embeddedness by analysing how individual resources (social, financial, or cultural capital; history of migration), markets (at the local, regional, and national levels), and political-institutional factors in the UK and overseas influence the development of transnational migrant entrepreneurs’ activities. This research area sheds new light on the complex interactions linking entrepreneurs and places, thus encouraging researchers to move beyond simple host-home country dichotomies.

Using geographical approaches, other authors examine the territories in which transnational entrepreneurs operate (Lan & Zhu, 2014; Zack, 2015). Some are critical of transnationalism approaches, arguing that a focus on abstract notions of flows ignores geographical locations (Mavrommatis, 2015) and preferring to speak of translocalism as a means for individuals to engage materially and symbolically with various sites through daily activities (Schmoll, 2012; Zani, 2019). These authors build on Brickell and Datta’s (2011) geographical concept of “translocality”, a key contribution to transnational mobilities studies. Translocality is for them “grounded transnationalism” (p. 3), or a space where otherwise de-territorialised networks of transnational social relations take shape through migrants’ agency. They argue for a spatial understanding of translocality, which situates migrants’ experiences within or across particular “locales” and does not confine them to the territorial boundaries of a nation-state. Furthermore, they bring the geographical scale to the fore by empirically addressing a range of scales including translocal homes, translocal neighbourhoods, and translocal cities.

By emphasising the role of agency and geographical position, these authors propose a powerful research lens through which to examine the experiences and practices of transnational migrant entrepreneurs. However, none of the current conceptual frameworks adequately address the complexity of how different locations provide different types of opportunities for transnational migrant entrepreneurs, and how they are able (or unable) to actively take advantage of such opportunities over space and time.

## 5 Towards a Spatially-Sensitive Approach in Future Research: Locations, Connections, and Mobilities

**Figure 1:**
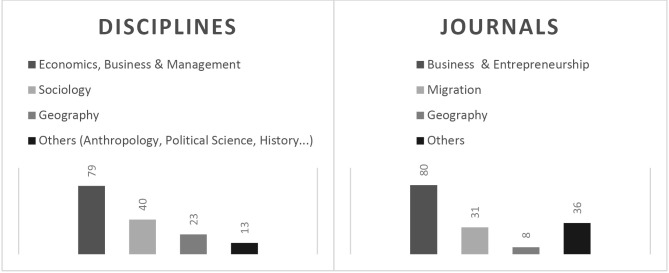
Overview of the disciplinary orientation of transnational migrant entrepreneurship studies published in scientific journals between 2009 and 2019, n=155

Our literature review reveals a dynamic field of cross-disciplinary transnational migrant entrepreneurship research. Currently, there appears to be a genuine attempt to shift the focus from a single-location and deficit-based perspective to an opportunity-oriented and transnational approach. This literature addresses important topics for geographers and other scholars interested in spatial dynamics. Yet, geographic contributions on transnational migrant entrepreneurship remain scarce, as seen in Figure 1.

Besides the willingness to move beyond simplistic and static conceptualisations of space in the field, there is much potential to develop a more spatially informed understanding of transnational migrant entrepreneurship, which would benefit geography research as well as other disciplines by enabling a greater focus on* the dynamic spatialities of transnational migrant entrepreneurship* and the *power relations that shape opportunities for different groups of entrepreneurs*. We will now discuss these gaps in more detail.

**Figure 2:**
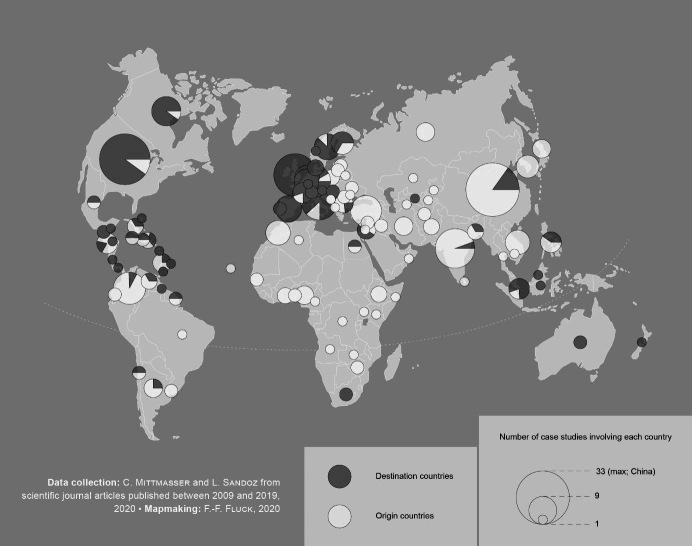
Origin and destination countries of migrants in transnational migrant entrepreneurship studies published in scientific journals between 2009 and 2019, n=155

First, the range of *locations* selected by scholars for empirical work on transnational migrant entrepreneurship is, in most cases, limited to countries of the Global North. This results in most studies focusing on the destination countries of transnational entrepreneurs with migratory trajectories from the Global South to the North. Figure 2 shows that almost 70 % of the articles in our study corpus focus on migrants who move from low-income countries in Asia, Africa, and South America to high-income countries in Europe or North America. This reproduces traditional representations of transnational migrant entrepreneurship as an exclusively South-to-North trajectory and downplays the diversity of current mobilities including South-to-South trajectories. Some scholars expand this view by examining migrants who move from high-income countries to low-income countries (Elo, 2016; Harima & Vermuri, 2015) or between two high-income countries (Lundberg & Rehnfors, 2018). However, the richness of South-to-South mobility patterns is still largely overlooked. We thus call for more attention to the diversity of transnational migrant entrepreneurs’ spatial trajectories.

Second, there is scant research on the diversity of *connections* that transnational migrant entrepreneurs create between locations across national borders. Most research on transnational migrant entrepreneurship examines connections between destination and home countries. Yet, as pointed out in a study on Moroccan entrepreneurs in Milan (Solano, 2020), transnational migrant entrepreneurship today is characterised by “multifocality” rather than “bifocality”. By disregarding the diversity of existing connections, authors reproduce a simplistic view of migration that does not correspond to contemporary transnationalism characterised by multisitedness and resulting from onward movements (Moret, 2018), facilitated international travel, lower transportation costs, digital technologies, and the development of transnationally located families (Riaño, 2017). Although transnational migrant entrepreneurs are, to a certain extent, conditioned by their nationalities and migration backgrounds, they can potentially use their agency to develop ties beyond national or ethnic groups, and connect with multiple places through entrepreneurial practices. Therefore, we need to expand our understanding of how transnational migrant entrepreneurs develop connections with multiple locations and create social, economic, and political relationships.

Third, insufficient attention has been paid to the diverse *mobilities* developed by transnational migrant entrepreneurs. Many studies have shown that engaging in transnational business activities requires travelling and/or moving goods, capital, or ideas across national borders (Ambrosini, 2012; Dannecker & Cakir, 2016). While spatial mobility is an essential dimension of transnational migrant entrepreneurship, studies that focus on how circulation patterns shape transnational entrepreneurial spaces remain scarce. In order to gain a better understanding of the spatialities of transnational migrant entrepreneurship, we propose applying a mobility lens (Cresswell, 2010; Sheller & Urry, 2006) to seriously study if, when, where, and how transnational migrant entrepreneurs move, and across which borders. This involves paying specific attention to the multi-scalar movements of transnational migrant entrepreneurs (e. g. home, neighbourhood, city, region, international), the different material means used to move (e. g. telephone, computer, streets, cars, trains, boats, planes), and the diversity of mobility patterns embodied (e. g. linear, circular, cyclical, pendular, onward, repeated returns). Moreover, in our technologically connected world, transnational business activities increasingly involve moving goods, ideas, and capital via digital means, which reduces the need for the spatial mobility of entrepreneurs (Harima & Baron, 2020). Focusing on the complexity of mobility and immobility patterns involved in transnational migrant entrepreneurship would not only deepen our understanding of how entrepreneurs create transnational spaces but also challenge static and ethnic biases in the field.

Finally, it is important to examine the diverse spatialities involved in transnational migrant entrepreneurship from a *power relations perspective* to understand how such spatialities relate to social inequality. Particularly in the business and management fields, authors tend to overemphasise the idea that transnational linkages constitute a competitive advantage for economic success without considering that structural inequalities create different geometries of power among transnational migrant entrepreneurs and thus different opportunities to benefit from transnational resources. Zapata-Barrero and Rezaei (2020) accurately highlight that most typologies of transnational migrant entrepreneurship are based on motivations and social status but ignore dimensions of inequality such as access to mobility, space, and territory. Opportunities for transnational migrant entrepreneurs to mobilise skills are not context-neutral but depend on how specific markers of social difference (e. g. gender, nationality, or education) are valued in the locations where they develop business activities. Also, inequalities of power between locations in rich and poor countries, and their impact on transnational migrant entrepreneurship, need further examination. Different countries implement different policies and agendas to attract, promote or discourage transnational migrant entrepreneurship (Tung, 2008). Power relations and mobility regimes shape hierarchies of opportunities and constraints for different categories of transnational migrant entrepreneurs (Sandoz, 2021).

Overall, there is great potential to move beyond static or ethnic framings of transnational migrant entrepreneurship and to strengthen our understanding of this topic. From a conceptual perspective, this could mean deeper engagement with the diversity of spatialities involved, as well as examining emerging webs of *geographical locations*, *connections*, and* mobilities*. From a methodological perspective, this could mean diversifying the current range of approaches to include digital (Kozinets, 2020), mobile (Boas et al., 2020), and multi-sited (Marcus, 1998) methods in future research.

Figure 3: Crucial dimensions for future research on the spatialities of transnational migrant entrepreneurship

***Dimension 1: Geographical locations***

Across/in which different locations do transnational migrant entrepreneurs move as they produce, obtain, and circulate goods, money, and services?
How do the material, social, and symbolic features of places shape the transnational business activities of entrepreneurs with diverse social backgrounds, and why? How do these features contribute to the (re)production of social inequalities?
How do entrepreneurs use different global locations for business activities and why? How do they transform the locations and shape the entrepreneurial ecosystems in which they operate?

***Dimension 2: Spatial connections***

What kinds of cross-border connections are created, developed, and maintained by transnational migrant entrepreneurs? Between which places and social groups do they build these connections?
What connections already exist between spaces and social groups (e. g. political, economic, or symbolic dependencies and hierarchies on the macro and micro levels)? How do these relations shape transnational businesses activities?
How do transnational entrepreneurs use and transform existing relations, and create new ones? What are the socioeconomic implications of the transnational connections established by transnational entrepreneurs?

***Dimension 3: Spatial mobilities***

What kinds of spatial mobilities are involved in transnational migrant entrepreneurship? How important is spatial mobility for transnational businesses? For whom is it important, and why?
Which spatial mobilities are possible for whom, under what conditions, and why? How do transnational migrant entrepreneurs’ capacities to move material and immaterial entities across time and space vary? How do these capacities evolve, and why?
How do entrepreneurs’ strategies counteract undesired mobilities or other constraints?

To go one step further, we propose some examples of questions for each of the three dimensions put forth in our analysis (locations, connections, and mobilities) to examine contemporary spatialities of migrant entrepreneurship (Figure 3). These questions arise from our critical literature review and build on the main shortcomings of current research, which we identified.

## 6 Conclusion

In this article we critically reviewed the literature on transnational migrant entrepreneurship published since 2009. We observed an increasing interest in this topic across a broad range of disciplines. Scholars have intensely studied the business advantages, characteristics, and networks of transnational migrant entrepreneurs as well as their impacts on specific sites (and vice versa). Yet, we observe that the new spatialities of transnational migrant entrepreneurship have not yet received adequate scholarly attention.

To address this gap, we proposed three crucial dimensions for future research which have been under-researched in past and current work, and which address the diversity of *geographical* *locations*, *spatial connections*, and *spatial mobilities* involved in transnational migrant entrepreneurship. We also advanced the following reflections: first, we argued that a processual and relational approach is necessary to understand how transnational entrepreneurial spaces are dynamically created and maintained through the everyday practices of individuals. Although geographers have so far marginally contributed to the field of transnational migrant entrepreneurship, the “relational turn” in economic geography (Bathelt & Glückler, 2003; 2018; Yeung, 2003) has addressed many of the shortcomings that we identified in this article and could therefore provide valuable tools for advancing our spatial perspective on this topic. Second, we encouraged researchers to include more diverse and multi-sited approaches to better grasp the diversity of the spatialities involved, particularly regarding transnational migrant entrepreneurship in the Global South and connections beyond the host and home countries of the studied entrepreneurs. Third, we suggested that in addition to empowering views that shed a positive light on transnational migrant entrepreneurship, more research is needed that addresses persistent social inequalities. It is important to ask how power relations between and across geographical locations and social groups shape the practices of entrepreneurs. In particular, the role of spatial mobility and immobility—and their relationship with social mobility—has not yet been sufficiently addressed in research on transnational migrant entrepreneurship.

In terms of future research, the discussion on how social mobility relates to transnational migrant entrepreneurship is immature but highly promising. Highlighting the role of locations, connections, and mobilities is important, not only to grasp the agency and creative potential of migrants but also to recognise that opportunities and constraints are unequally distributed across social groups and places. We need to advance our understanding of unequal spatialities in transnational migrant entrepreneurship and identify the mechanisms that enable individual and family businesses to move forward economically and socially. This is important not only for research, but also in the development of more inclusive places for migrant entrepreneurs, which could lead to better opportunities for success.
