# Supplementary material for: A Review of Transnational Migrant Entrepreneurship: Perspectives on Unequal Spatialities
Source: ZFW Adv Econ Geogr. 2022 Oct 11;66(3):137–50. doi: 10.1515/zfw-2021-0004 (PMC9612877; doi:10.1515/zfw-2021-0004)
Supplement: Supplementary file 1 [file Online_Appendix_A_Review_of_Transnational_Migrant_Entrepreneurship.pdf]

Online Appendix

A Review of Transnational Migrant Entrepreneurship: Perspectives on Unequal Spatialities

Laure Sandoz, Christina Mittmasser, Yvonne Riaño, Etienne Piguet

https://doi.org/10.1515/zfw-2021-0004 - Supplementary material

1. Main databases and journals monitored for the automated literature search

Resource name	Resource type	Alert type
Scopus	Database	RSS
Elsevier ScienceDirect	Database	Email
Sage Journals	Database	Email
Springer Link	Database	RSS
Taylor and Francis	Database	Email
Wiley Journals	Database	Email
Google scholar	Research engine	Email
ABI/INFORM Complete (+ Econlit)	Database	RSS via Proquest
Business Source Premier	Database	RSS
Emerald Management Xtra	Database	Email
OECD	Database	RSS/Update scanner
IBSS	Database	RSS via Proquest
SocIndex	Database	RSS
Base (Bielefeld Academic Search Engine)	Database	RSS
Work Employment & Society	Journal	Feed Informer
Entrepreneurship & Regional Development	Journal	Feed Informer
International Migration Review	Journal	Feed Informer
International migration	Journal	Feed Informer
Migration Studies	Journal	Email
Journal of ethnic and migration studies	Journal	Feed Informer
Ethnic & racial studies	Journal	Feed Informer
Global networks	Journal	Feed Informer
Social networks	Journal	Feed Informer
Journal of international entrepreneurship	Journal	Feed Informer
Entrepreneurship: Theory and Practice	Journal	Feed Informer
Entrepreneurship and regional development	Journal	Feed Informer
International Journal of Entrepreneurship and small business	Journal	Feed Informer
Revue européenne des migrations internationales	Journal	Feed Informer
Journal of international migration and integration	Journal	Feed Informer
Cuadernos de investigacion geografica	Journal	Feed Informer + email
Papers : revista de sociologia	Journal	Feed Informer
Migraciones internacionales	Journal	Google alert
Estudios geograficos	Journal	Feed Informer
Revista espanola de sociologia	Journal	email
Culture & conflits	Journal	Feed Informer
Oesterreichische Zeitschrift für Soziologie	Journal	Feed Informer
Raumforschung und Raumordnung	Journal	Feed Informer
International journal of entrepreneurial behavior & research	Journal	Feed Informer

2. Main keywords used for the literature search

English	French	German	Spanish
Transnational OR transnationalism<br>AND Migration OR Migrant OR Immigrant<br>AND Entrepreneur OR entrepreneurship OR business OR start-up OR firm	Transnational OR transnationalisme<br>AND Migration OR migrant OR immigré OR étranger<br>AND Entrepreneur OR entreprenariat OR entrepreneuriat OR entreprise OR commerce OR start-up	Transnational OR transnationalismus<br>AND Migration OR Migrant OR Einwanderer OR Einwanderung<br>AND Unternehmer OR Unternehmertum OR Entrepreneur OR selbständig OR Unternehmen OR Start-up	Transnacional OR transnacionalismo<br>AND migración OR inmigrante OR migrante<br>AND empresario OR emprendedor OR empresariado OR emprendimiento OR empresa OR comercio OR start-up

3. Selection of articles included in the literature review (n=155)

1. Åkesson, L. (2016) Multi-sited accumulation of capital: Cape Verdean returnees and small-scale business. *Global Networks*, 16(1): 112–129. https://doi.org/10.1111/glob.12100
2. Ambrosini, M. (2012) Migrants' entrepreneurship in transnational social fields: Research in the Italian context. *International Review of Sociology*, 22(2): 273–292. https://www.tandfonline.com/doi/full/10.1080/03906701.2012.696970
3. Ambrosini, M. (2014) Migration and transnational commitment: Some evidence from the Italian case. *Journal of Ethnic and Migration Studies*, 40(4): 619–637. https://doi.org/10.1080/1369183X.2013.830883
4. Ashourizadeh, S., Schøtt, T., Şengüler, E. P., & Wang, Y. (2016) Exporting by migrants and indigenous entrepreneurs: Contingent on gender and education. *International Journal of Business and Globalisation*, 16(3): 264–283. https://doi.org/10.1504/IJBG.2016.075736
5. Bagwell, S. (2015) Transnational entrepreneurship amongst Vietnamese businesses in London. *Journal of Ethnic and Migration Studies*, 41(2): 329–349. https://doi.org/10.1080/1369183X.2014.907739

6. Bagwell, S. (2018) From mixed embeddedness to transnational mixed embeddedness: An exploration of Vietnamese businesses in London. *International Journal of Entrepreneurial Behavior & Research*, 24(1): 104–120. https://doi.org/10.1108/IJEBR-01-2017-0035
7. Baklanov, N., Rezaei, S., Brambini-Pedersen, J. V., & Dana, L.-P. (2014) Migrant entrepreneurship, economic activity and export performance: Mapping the Danish trends. *International Journal of Entrepreneurship and Small Business*, 23(1/2): 63–93. https://doi.org/10.1504/IJESB.2014.065309
8. Baltar, F., & Icart, I. B. (2013) Entrepreneurial gain, cultural similarity and transnational entrepreneurship. *Global Networks*, 13(2): 200–220. https://doi.org/10.1111/glob.12020
9. Barberis, E., & Solano, G. (2018) Mixed embeddedness and migrant entrepreneurship: Hints on past and future directions. An introduction. *Sociologica*, 12(2): 1–22. https://doi.org/10.6092/issn.1971-8853/8617
10. Baycan, T. (2013) Turkish Entrepreneurship in Europe. *European Review*, 21(3): 382–393. https://doi.org/10.1017/S1062798713000343
11. Bloh, J. von, Mandakovic, V., Apablaza, M., Amorós, J. E., & Sternberg, R. (2019) Transnational entrepreneurs: Opportunity or necessity driven? Empirical evidence from two dynamic economies from Latin America and Europe. *Journal of Ethnic and Migration Studies*. https://doi.org/10.1080/1369183X.2018.1559996
12. Brzozowski, J., Cucculelli, M., & Surdej, A. (2014) Transnational ties and performance of immigrant entrepreneurs: The role of home-country conditions. *Entrepreneurship & Regional Development*, 26(7–8): 546–573. https://doi.org/10.1080/08985626.2014.959068
13. Brzozowski, J., Cucculelli, M., & Surdej, A. (2017) The determinants of transnational entrepreneurship and transnational ties' dynamics among immigrant entrepreneurs in ICT sector in Italy. *International Migration*, 55(3): 105–125. https://doi.org/10.1111/imig.12335
14. Brzozowski, J., Cucculelli, M., & Surdej, A. (2019) Exploring transnational entrepreneurship. Immigrant entrepreneurs and foreign-born returnees in the Italian ICT sector. *Journal of Small Business & Entrepreneurship*, 31(5): 413–431. https://doi.org/10.1080/08276331.2018.1429803
15. Chen, W., & Tan, J. (2009) Understanding transnational entrepreneurship through a network lens: Theoretical and methodological considerations. *Entrepreneurship Theory and Practice*, 33(5): 1079–1091. https://doi.org/10.1111/j.1540-6520.2009.00335.x
16. Chou, T. L., Ching, C.-H., Fan, S., & Chang, J.-Y. (2011) Global linkages, the Chinese high-tech community and industrial cluster development: The semiconductor industry in Wuxi, Jingsu. *Urban Studies*, 48(14): 3019–3042. https://doi.org/10.1177/0042098010396237

17. Colic-Peisker, V., & Deng, L. (2019) Chinese business migrants in Australia: Middle-class transnationalism and 'dual embeddedness'. *Journal of Sociology*, 55(2): 234–251. https://doi.org/10.1177/1440783319836281
18. Crick, D., & Chaudhry, S. (2010) An investigation into UK-based Asian entrepreneurs' perceived competitiveness in overseas markets. *Entrepreneurship & Regional Development*, 22(1): 5–23. https://doi.org/10.1080/08985620903220520
19. Crick, D., & Chaudhry, S. (2013) An exploratory study of UK based, family-owned, Asian firms' motives for internationalising. *Journal of Small Business and Enterprise Development*, 20(3): 526–547. https://doi.org/10.1108/JSBED-04-2013-0051
20. Cucculelli, M., & Morettini, G. (2012) Transnational ties in technology-based sectors: The case of Indian software entrepreneurial firms in Italy. *International Journal of Entrepreneurship and Small Business*, 17(3): 319. https://doi.org/10.1504/IJESB.2012.049580
21. Dahles, H. (2013) Cambodian returnees' entrepreneurial ventures: The opportunities and constraints of mixed embeddedness. *Journal of Enterprising Communities: People and Places in the Global Economy*, 7(4): 383–396. https://doi.org/10.1108/JEC-03-2013-0009
22. De Luca, D., & Ambrosini, M. (2019) Female immigrant entrepreneurs: More than a family strategy. *International Migration*, 57(5): 201–215. https://doi.org/10.1111/imig.12564
23. de Silva, R. (2015) How transnational entrepreneurs are responding to institutional voids: A study of transnational entrepreneurs from Sri Lanka. *South Asian Journal of Management*, 22(2): 61–80.
24. Dimitratos, P., Buck, T., Fletcher, M., & Li, N. (2016) The motivation of international entrepreneurship: The case of Chinese transnational entrepreneurs. *International Business Review*, 25: 1103–1113. https://doi.org/10.1016/j.ibusrev.2016.01.012
25. Drori, I., Honig, B., & Wright, M. (2009) Transnational entrepreneurship: An emergent field of study. *Entrepreneurship Theory and Practice*, 33(5): 1001–1022. https://doi.org/10.1111/j.1540-6520.2009.00332.x
26. Eckstein, S., & Nguyen, T.-N. (2011) The making and transnationalization of an ethnic niche: Vietnamese manicurists. *The International Migration Review*, 45(3): 639–674. https://doi.org/10.1111/j.1747-7379.2011.00861.x
27. Eimermann, M., & Karlsson, S. (2018) Globalising Swedish countrysides? A relational approach to rural immigrant restaurateurs with refugee backgrounds. *Norwegian Journal of Geography*, 72(2): 82–96. https://doi.org/10.1080/00291951.2018.1450781
28. Elo, M. (2016) Typology of diaspora entrepreneurship: Case studies in Uzbekistan. *Journal of International Entrepreneurship*, 14(1): 121–155. https://doi.org/10.1007/s10843-016-0177-9

29. Elo, M. (2019) Immigrant effect and collective entrepreneurship—The creation and development of a Turkish entrepreneurial group. *Historical Social Research*, 44(4): 129–161. https://doi.org/10.12759/hsr.44.2019.4.129-161
30. Elo, M., & Freiling, J. (2015) Transnational Entrepreneurship: An Introduction to the Volume. *American Journal of Entrepreneurship*, 8(2).
31. Elo, M., & Hieta, H. (2017) From ethnic enclaves to transnational entrepreneurs: The American dream of the Finns in Oregon, USA. *International Journal of Entrepreneurship and Small Business*, 31(2): 204–226.
32. Elo, M., & Jokela, P. (2015) Transnational entrepreneurship among Bukharian Jewish diaspora in Israel. *American Journal of Entrepreneurship*, 8(2): 22–42.
33. Elo, M., Sandberg, S., Servais, P., Basco, R., Cruz, A. D., & Täube, F. (2018) Advancing the views on migrant and diaspora entrepreneurs in international entrepreneurship. *Journal of International Entrepreneurship*, 16(2): 119–133. https://doi.org/10.1007/s10843-018-0231-x
34. Elo, M., & Vincze, Z. (2019) Transnational intrapreneurship: Opportunity development in transnational teams in the Nordic periphery. *International Journal of Entrepreneurship and Small Business*, 36(1–2): 103–125. https://doi.org/10.1504/IJESB.2019.096954
35. Emontspool, J., & Servais, P. (2017) Cross-border entrepreneurship in a global world: A critical reconceptualisation. *European Journal of International Management*, 11(3): 262–279. https://doi.org/10.1504/EJIM.2017.083875
36. Evansluong, Q., Ramírez Pasillas, M., Discua Cruz, A., Elo, M., & Vershinina, N. (2019) Migrant entrepreneurship beyond place and space: A call to explore the roles of family across borders and contexts. *Journal of Enterprising Communities: People and Places in the Global Economy*. http://lup.lub.lu.se/record/9572d00c-e5cc-422c-9dcf-ca2b3487cdff
37. Fossati, D. (2019) Embedded diasporas: Ethnic prejudice, transnational networks and foreign investment. *Review of International Political Economy*, 26(1): 134–157. https://doi.org/10.1080/09692290.2018.1543721
38. Fuller, D. B. (2010) How law, politics and transnational networks affect technology entrepreneurship: Explaining divergent venture capital investing strategies in China. *Asia Pacific Journal of Management*, 27(3): 445–459. https://doi.org/10.1007/s10490-009-9149-0
39. Fürst, A., & Balke, J. (2013) Transnationales ethnisches Unternehmertum. Das Fallbeispiel türkischstämmiger Unternehmer in Duisburg-Marxloh. *Raumforschung und Raumordnung*, 71(3): 247–259. https://doi.org/10.1007/s13147-013-0233-z
40. Gangadhar, N., & Manohar, B. M. (2015) Understanding transnational entrepreneurship. *International Journal of Advances in Management and Economics*, 4(6): 52–67.
41. Garrido, A. A., & Checa, J. C. (2009) Theoretical overview of immigrant entrepreneurship. *Sociologica*, 41(2): 199–221.

42. Glick Schiller, N., & Çağlar, A. (2013) Locating migrant pathways of economic emplacement: Thinking beyond the ethnic lens. *Ethnicities*, 13(4): 494–514. https://doi.org/10.1177/1468796813483733
43. Glorius, B. (2019) Transnational social capital in migration: The example of educational migration between Bulgaria and Germany. *Social Inclusion*, 7(4): 232–242. https://doi.org/10.17645/si.v7i4.2390
44. Goktan, B., & Flores, I. B. (2014) The role of national culture in the transnational entrepreneurship process. *Journal of Business and Entrepreneurship*, 25(2): 75–95.
45. Halilovich, H., & Efendić, N. (2019) From refugees to trans-local entrepreneurs: Crossing the borders between formal institutions and informal practices in Bosnia and Herzegovina. *Journal of Refugee Studies*. https://doi.org/10.1093/jrs/fey066
46. Harima, A. (2014) Network dynamics of descending diaspora entrepreneurship: Multiple case studies with Japanese entrepreneurs in emerging economies. Journal of Entrepreneurship, *Management and Innovation*, 10(4): 65–92.
47. Harima, A., & Vermuri, D. S. (2015) Diaspora business model innovation. Journal of Entrepreneurship, *Management and Innovation*, 11(1): 29–52. https://doi.org/10.7341/20151113
48. Henn, S. (2011) Transnationale Unternehmer und die Entstehung von "Southern Multinationals". Das Fallbeispiel indischer Diamanthändler. *Geographische Zeitschrift*, 99(4): 202–219.
49. Henn, S. (2012) Transnational entrepreneurs, global pipelines and shifting production patterns. The example of the Palanpuris in the diamond sector. *Geoforum*, 43(3): 497–506. https://doi.org/10.1016/j.geoforum.2011.10.009
50. Henn, S. (2013) Transnational entrepreneurs and the emergence of clusters in peripheral regions. The case of the diamond cutting cluster in Gujarat (India). *European Planning Studies*, 21(11): 1779–1795. https://doi.org/10.1080/09654313.2012.753690
51. Honig, B. (2019) Exploring the intersection of transnational, ethnic, and migration entrepreneurship. *Journal of Ethnic and Migration Studies*. https://doi.org/10.1080/1369183X.2018.1559993
52. Ilhan-Nas, T., Sahin, K., & Cilingir, Z. (2011) International ethnic entrepreneurship: Antecedents, outcomes and environmental context. *International Business Review*, 20(6): 614–626. https://doi.org/10.1016/j.ibusrev.2011.02.011
53. Jensen, K. W., Rezaei, S., & Wherry, F. F. (2014) Cognitive effects on entrepreneurial intentions: A comparison of Chinese émigrés and their descendants with non-émigré Chinese. *International Journal of Entrepreneurship and Small Business*, 23(1/2): 252. https://doi.org/10.1504/IJESB.2014.065304

54. Jones, T., Ram, M., & Theodorakopoulos, N. (2010) Transnationalism as a force for ethnic minority enterprise? The case of Somalis in Leicester. *International Journal of Urban and Regional Research*, 34(3): 565–585. https://doi.org/10.1111/j.1468-2427.2010.00913.x
55. Kariv, D., Menzies, T. V., Brenner, G. A., & Filion, L. J. (2009) Transnational networking and business performance: Ethnic entrepreneurs in Canada. *Entrepreneurship & Regional Development*, 21(3): 239–264. https://doi.org/10.1080/08985620802261641
56. Katila, S., & Wahlbeck, Ö. (2012) The role of (transnational) social capital in the start-up processes of immigrant businesses: The case of Chinese and Turkish restaurant businesses in Finland. *International Small Business Journal*, 30(3): 294–309. https://doi.org/10.1177/0266242610383789
57. Kuah-Pearce, K. E. (2016) Migrant women entrepreneurs in the garment industry in modern China: Embedding translocality and feminised Guanxi networks. *International Journal of Business and Globalisation*, 16(3): 335. https://doi.org/10.1504/IJBG.2016.075730
58. Kwak, M.-J., & Hiebert, D. (2010) Globalizing Canadian education from below: A case study of transnational immigrant entrepreneurship between Seoul, Korea and Vancouver Canada. *Journal of International Migration and Integration*, 11(2): 131–153. https://doi.org/10.1007/s12134-010-0130-z
59. Lan, T., & Zhu, S. (2014) Chinese apparel value chains in Europe: Low-end fast fashion, regionalization, and transnational entrepreneurship in Prato, Italy. *Eurasian Geography and Economics*, 55(2): 156–174. https://doi.org/10.1080/15387216.2014.948471
60. Lee, J. Y., & Lee, J. Y. (2019) Female transnational entrepreneurs (FTEs): A case study of Korean American female entrepreneurs in Silicon Valley. *Journal of Entrepreneurship and Innovation in Emerging Economies*. https://doi.org/10.1177/2393957519881925
61. Ley, D. (2013) Does Transnationalism Trump Immigrant Integration? Evidence from Canada's Links with East Asia. *Journal of Ethnic and Migration Studies*, 39(6): 921–938. https://doi.org/10.1080/1369183X.2013.765654
62. Li, Z. (2018) La diaspora entrepreneuriale Wenzhou en région parisienne: Une diaspora parmi les diasporas chinoises. *Migrations Société*, 174(4): 123–139.
63. Light, I. (2014) Transnationals in an English-speaking world. *International Journal of Entrepreneurship and Small Business*, 23(1–2): 10–26. https://doi.org/10.1504/IJESB.2014.065307
64. Light, I., & Shahlapour, P. (2016) Transnational Iranian entrepreneurs in the import/export industry of Los Angeles. *International Journal of Business and Globalisation*, 16(3): 304. https://doi.org/10.1504/IJBG.2016.075732

65. Lin, X., & Tao, S. (2012) Transnational entrepreneurs: Characteristics, drivers, and success factors. *Journal of International Entrepreneurship*, 10(1): 50–69. https://doi.org/10.1007/s10843-011-0082-1
66. Liu, H. (2012) Beyond a revisionist turn: Networks, state and the changing dynamics of diasporic Chinese entrepreneurship. *China An International Journal*, 10(3): 20.41.
67. Liu, Y., Namatovu, R., Karadeniz, E. E., Schøtt, T., & Minto-Coy, I. D. (2019) Entrepreneurs' transnational networks channelling exports: Diasporas from Central & South America, Sub-Sahara Africa, Middle East & North Africa, Asia, and the European culture region. *Journal of Ethnic and Migration Studies*. https://doi.org/10.1080/1369183X.2018.1560002
68. Lundberg, H., & Rehnfors, A. (2018) Transnational entrepreneurship: Opportunity identification and venture creation. *Journal of International Entrepreneurship*, 16(2): 150–175. https://doi.org/10.1007/s10843-018-0228-5
69. Ma, Z., Zhao, S., Wang, T., & Lee, Y. (2013) An overview of contemporary ethnic entrepreneurship studies: Themes and relationships. International *Journal of Entrepreneurial Behavior & Research*, 19(1): 32–52. https://doi.org/10.1108/13552551311299242
70. Maceda Rodriguez, E., & Vazquez, Vazquez, J. D. (2016) Transnacionalismo y rasgos de desarrollo local en Santa Inés Ahuatempan, Puebla. *Regiones y Desarrollo Sustentable*, 31: 133–153.
71. Mavrommatis, G. (2015) South Asian tales: Ethnic entrepreneurship and narratives of spatialized transnational identities emerging in an East London (UK), inner-city area. *Diaspora Studies*, 8(2): 89–103. https://doi.org/10.1080/09739572.2015.1029710
72. Moghaddam, K., Rustambekov, E., Weber, T., & Azarpanah, S. (2018) Transnational entrepreneurship, social networks, and institutional distance: Toward a theoretical framework. *New England Journal of Entrepreneurship*, 21(1): 45–64. https://doi.org/10.1108/NEJE-03-2018-0005
73. Mung, E. M. (2012) Migrations et transmigrations dans la diaspora entrepreneuriale chinoise. *Multitudes*, 49(2): 53–61.
74. Munkejord, M. C. (2017) Local and transnational networking among female immigrant entrepreneurs in peripheral rural contexts: Perspectives on Russians in Finnmark, Norway. *European Urban and Regional Studies*, 24(1): 7–20. https://doi.org/10.1177/0969776415587122
75. Muñoz Castro, D. C., Alvarez, S. M. S., & Zapata, S. I. M. (2019) Transnational entrepreneurship: A systematic review of the literature. *International Journal of Entrepreneurship and Innovation Management*, 23(6): 559–583. https://doi.org/10.1504/IJEIM.2019.102824

76. Mustafa, M., & Chen, S. (2010) The strength of family networks in transnational immigrant entrepreneurship. *Thunderbird International Business Review*, 52(2): 97–106. https://doi.org/10.1002/tie.20317
77. Nawojczyk, M., & Nowicka, M. (2018) Transnational migration and entrepreneurial activities of migrants. Introduction. *Migration Studies - Review of Polish Diaspora*, 169(3): 9–15.
78. Nawojczyk, M., & Synowiec-Jaje, L. (2018) Blurred borders. Entrepreneurial activity of Poles in Germany in the eyes of experts. *Studia Migracyjne – Przegląd Polonijny*, 3: 41–58. https://doi.org/10.4467/25444972SMPP.18.034.9433
79. Nkongolo-Bakenda, J.-M., & Chrysostome, E. V. (2013) Engaging diasporas as international entrepreneurs in developing countries: In search of determinants. *Journal of International Entrepreneurship*, 11(1): 30–64. https://doi.org/10.1007/s10843-012-0098-1
80. Nkrumah, A. (2018) Immigrants' transnational entrepreneurial activities: The case of Ghanaian immigrants in Canada. *Journal of International Migration and Integration*, 19(1): 195–211. https://doi.org/10.1007/s12134-017-0535-z
81. Nowicka, M. (2013) Positioning strategies of Polish entrepreneurs in Germany: Transnationalizing Bourdieu's notion of capital. *International Sociology*, 28(1): 29–47. https://doi.org/10.1177/0268580912468919
82. Nyíri, P. (2011) Chinese entrepreneurs in poor countries: A transnational 'middleman minority' and its futures. *Inter-Asia Cultural Studies*, 12(1): 145–153. https://doi.org/10.1080/14649373.2011.532985
83. Ojo, S. (2012) Ethnic enclaves to diaspora entrepreneurs: A critical appraisal of Black British Africans' transnational entrepreneurship in London. *Journal of African Business*, 13(2): 145–156. https://doi.org/10.1080/15228916.2012.693446
84. Ojo, S. (2017) Interrogating returnee entrepreneurship in the Nigerian context. *Journal of Enterprising Communities: People and Places in the Global Economy*, 11(5): 590–608. https://doi.org/10.1108/JEC-07-2016-0025
85. Ojo, S., Nwankwo, S., & Gbadamosi, A. (2013) African diaspora entrepreneurs: Navigating entrepreneurial spaces in 'Home' and 'Host' countries. *The International Journal of Entrepreneurship and Innovation*, 14(4): 289–299. https://doi.org/10.5367/ijei.2013.0126
86. Osaghae, O.-G., & Cooney, T. M. (2019) Exploring the relationship between immigrant enclave theory and transnational diaspora entrepreneurial opportunity formation. *Journal of Ethnic and Migration Studies*. https://doi.org/10.1080/1369183X.2018.1560001
87. Ottati, G. D. (2014) A transnational fast fashion industrial district: An analysis of the Chinese businesses in Prato. *Cambridge Journal of Economics*, 38(5): 1247–1274. https://doi.org/10.1093/cje/beu015

88. Ozasir Kacar, S., & Essers, C. (2019) The interplay between identity construction and opportunity structures: Narratives of Turkish migrant women entrepreneurs in the Netherlands. *International Small Business Journal*, 37(7): 713–731. https://doi.org/10.1177/0266242619856809
89. Patel, P. C., & Conklin, B. (2009) The balancing act: The Role of transnational habitus and social networks in balancing transnational entrepreneurial activities. *Entrepreneurship Theory and Practice*, 33(5): 1045–1078. https://doi.org/10.1111/j.1540-6520.2009.00334.x
90. Patel, P. C., & Terjesen, S. (2011) Complementary effects of network range and tie strength in enhancing transnational venture performance. *Strategic Entrepreneurship Journal*, 5(1): 58–80. https://doi.org/10.1002/sej.107
91. Pio, E., & Essers, C. (2014) Professional migrant women decentring otherness: A transnational perspective: Professional migrant women decentring otherness. *British Journal of Management*, 25(2): 252–265. https://doi.org/10.1111/1467-8551.12003
92. Poblete, C. (2018) Shaping the castle according to the rocks in the path? Perceived discrimination, social differences, and subjective wellbeing as determinants of firm type among immigrant entrepreneurs. *Journal of International Entrepreneurship*, 16(2): 276–300. https://doi.org/10.1007/s10843-018-0224-9
93. Portes, A., & Martinez, B. P. (2019) They are not all the same: Immigrant enterprises, transnationalism, and development. *Journal of Ethnic and Migration Studies*. https://doi.org/10.1080/1369183X.2018.1559995
94. Portes, A., & Yiu, J. (2013) Entrepreneurship, transnationalism, and development. *Migration Studies*, 1(1): 75–95. https://doi.org/10.1093/migration/mns036
95. Pruthi, S. (2014) Social ties and venture creation by returnee entrepreneurs. *International Business Review*, 23(6): 1139–1152. https://doi.org/10.1016/j.ibusrev.2014.03.012
96. Pruthi, S., Basu, A., & Wright, M. (2018) Ethnic ties, motivations, and home country entry strategy of transnational entrepreneurs. *Journal of International Entrepreneurship*, 16(2): 210–243. https://doi.org/10.1007/s10843-017-0223-2
97. Pruthi, S., & Wright, M. (2017) Social ties, social capital, and recruiting managers in transnational ventures. *Journal of East-West Business*, 23(2): 105–139. https://doi.org/10.1080/10669868.2016.1270247
98. Pruthi, S., & Wright, M. (2019) Social ties, prior experience, and venture creation by transnational entrepreneurs. *International Journal of Entrepreneurship and Small Business*, 36(1/2): 41–73. https://doi.org/10.1504/IJESB.2019.096964
99. Quan, R., Fan, M., Zhang, M., & Sun, H. (2019) A dynamic dual model: The determinants of transnational migrant entrepreneurs' embeddedness in the UK. Journal of Entrepreneurship, *Management and Innovation*, 15(2): 29–55. https://doi.org/10.7341/20191522

100. Rana, M. B., & Elo, M. (2017) Transnational diaspora and civil society actors driving MNE internationalisation: The case of Grameenphone in Bangladesh. *Journal of International Management*, 23(1): 87–106. https://doi.org/10.1016/j.intman.2016.11.005

101. Ren, N., & Liu, H. (2015) Traversing between transnationalism and integration: Dual embeddedness of new Chinese immigrant entrepreneurs in Singapore. *Asian and Pacific Migration Journal*, 24(3): 298–326. https://doi.org/10.1177/0117196815594719

102. Ren, N., & Liu, H. (2019) Domesticating 'transnational cultural capital': The Chinese state and diasporic technopreneur returnees. *Journal of Ethnic and Migration Studies*, 45(13): 2308–2327. https://doi.org/10.1080/1369183X.2018.1534583

103. Rezaei, S., & Goli, M. (2019) Prometheus, the double-troubled – migrant transnational entrepreneurs and the loyalty trap. *Journal of Ethnic and Migration Studies*. https://doi.org/10.1080/1369183X.2018.1559998

104. Rezaei, S., Light, I., & Telles, E. E. (2016) Brain Circulation and Transnational Entrepreneurship: Guest Editorial—Special Issue. *International Journal of Business and Globalisation*, 16(3): 203–208.

105. Ribeiro, A., Rezaei, S., & Dana, L.-P. (2012) Gender and family in transnational entrepreneurship. *International Journal of Business and Globalisation*, 8(3): 409–420. https://doi.org/10.1504/IJBG.2012.046213

106. Riddle, L., Hrivnak, G. A., & Nielsen, T. M. (2010) Transnational diaspora entrepreneurship in emerging markets: Bridging institutional divides. *Journal of International Management*, 16(4): 398–411. https://doi.org/10.1016/j.intman.2010.09.009

107. Rodgers, P., Vershinina, N., Williams, C. C., & Theodorakopoulos, N. (2019) Leveraging symbolic capital: The use of *blat* networks across transnational spaces. *Global Networks*, 19(1): 119–136. https://doi.org/10.1111/glob.12188

108. Rogerson, J. M., & Mushawemhuka, W. J. (2015) Transnational entrepreneurship in the Global South: Evidence from Southern Africa. *Bulletin of Geography. Socio-Economic Series*, 30(30): 135–146. https://doi.org/10.1515/bog-2015-0040

109. Rosenfeld, M. (2012) Mobility and social capital among Lebanese and Beninese entrepreneurs engaged in transnational trade. *International Review of Sociology*, 22(2): 211–228. https://www.tandfonline.com/doi/full/10.1080/03906701.2012.696962

110. Rosenfeld, M. (2013) Entrepreneurs transnationaux et commerce d'exportation de véhicules d'occasion: La filière Bruxelles-Cotonou. *Revue européenne des migrations internationales*, 29(2) : 57–76. https://doi.org/10.4000/remi.6399

111. Sandberg, S., Immonen, R., & Kok, S. (2018) Refugee entrepreneurship: Taking a social network view on immigrants with refugee backgrounds starting transnational businesses in

Sweden. *International Journal of Entrepreneurship and Small Business*, 36(1–2): 216–241. https://doi.org/10.1504/IJESB.2019.096967

112. Santamaria-Alvarez, S. M., Muñoz-Castro, D. C., Sarmiento-González, M. A., & Marín-Zapata, S. I. (2018) Fragmented networks and transnational entrepreneurship: Building strategies to prosper in challenging surroundings. *Journal of International Entrepreneurship*, 16(2): 244–275. https://doi.org/10.1007/s10843-017-0215-2

113. Santamaria-Alvarez, S. M., Sarmiento-González, M. A., & Arango-Vieira, L. C. (2019) Transnational migrant entrepreneur characteristics and the transnational business nexus: The Colombian case. *International Journal of Entrepreneurial Behavior & Research*, 25(5): 1014–1044. https://doi.org/10.1108/IJEBR-02-2018-0092

114. Santamaria-Alvarez, S. M., & Śliwa, M. (2016a) Transnational activities of Colombians in the US. *Critical Perspectives on International Business*, 12(2): 140–166. http://dx.doi.org/10.1108/cpoib-09-2012-0038

115. Santamaria-Alvarez, S. M., & Śliwa, M. (2016b) Transnational entrepreneurship in emerging markets: The Colombian case. *Journal of Enterprising Communities: People and Places in the Global Economy*, 10(2): 203–223. https://doi.org/10.1108/JEC-10-2013-0030

116. Schäfer, S., & Henn, S. (2018) The evolution of entrepreneurial ecosystems and the critical role of migrants. A Phase-Model based on a Study of IT startups in the Greater Tel Aviv Area. *Cambridge Journal of Regions, Economy and Society*, 11(2): 317–333. https://doi.org/10.1093/cjres/rsy013

117. Schmoll, C. (2012) The making of a transnational marketplace. Naples and the impact of Mediterranean cross-border trade on regional economies. *Cambridge Journal of Regions, Economy and Society*, 5(2): 221–238. https://doi.org/10.1093/cjres/rsr020

118. Schøtt, T. (2018) Entrepreneurial pursuits in the Caribbean diaspora: Networks and their mixed effects. *Entrepreneurship & Regional Development*, 30(9–10): 1069–1090. https://doi.org/10.1080/08985626.2018.1515825

119. Sequeira, J. M., Carr, J. C., & Rasheed, A. A. (2009) Transnational entrepreneurship: Determinants of firm type and owner attributions of success. *Entrepreneurship Theory and Practice*, 33(5): 1023–1044. https://doi.org/10.1111/j.1540-6520.2009.00333.x

120. Shin, K.-H. (2014) Korean entrepreneurs in Kansas City metropolitan area: An immigrant community under ethnic local and global intersection. *Journal of Enterprising Communities: People and Places in the Global Economy*, 8(4): 287–299. https://doi.org/10.1108/JEC-11-2013-0034

121. Sinatti, G. (2018) Return migration, entrepreneurship and development. *African Studies*, 38(4): 1–15. https://doi.org/10.1080/00020184.2018.1555310

122. Smallbone, D., Kitching, J., & Athayde, R. (2010) Ethnic diversity, entrepreneurship and competitiveness in a global city. *International Small Business Journal*, 28(2): 174–190. https://doi.org/10.1177/0266242609355856

123. Solano, G. (2015) Transnational vs. Domestic immigrant entrepreneurs: A comparative literature analysis of the use of personal skills and social networks. *American Journal of Entrepreneurship*, 8(2): 1–21.

124. Solano, G. (2016) Multifocal entrepreneurial practices: The case of Moroccan import/export businesses in Milan. *International Journal of Entrepreneurship and Small Business*, 29(2). https://doi.org/10.1504/IJESB.2016.078698

125. Solano, G. (2019) The mixed embeddedness of transnational migrant entrepreneurs: Moroccans in Amsterdam and Milan. *Journal of Ethnic and Migration Studies*. https://doi.org/10.1080/1369183X.2018.1559999

126. Sommer, E., & Gamper, M. (2018) Transnational entrepreneurial activities: A qualitative network study of self-employed migrants from the former Soviet Union in Germany. *Social Networks*, 53: 136–147. https://doi.org/10.1016/j.socnet.2017.04.007

127. Sternberg, R., & Mueller, C. (2010) "New argonauts" in China—Return migrants, transnational entrepreneurship and economic growth in a regional innovation system. *Die Erde; Zeitschrift Der Gesellschaft Für Erdkunde Zu Berlin*, 141: 103–125.

128. Storti, L. (2014) Being an entrepreneur: Emergence and structuring of two immigrant entrepreneur groups. *Entrepreneurship & Regional Development*, 26(7–8): 521–545. https://doi.org/10.1080/08985626.2014.959067

129. Stoyanov, S. (2018) Enabling social identity interaction: Bulgarian migrant entrepreneurs building embeddedness to a transnational network. *British Journal of Management*, 29(2): 373–388. https://doi.org/10.1111/1467-8551.12235

130. Stoyanov, S., Woodward, R., & Stoyanova, V. (2018) The embedding of transnational entrepreneurs in diaspora networks: Leveraging the assets of foreignness. *Management International Review*, 58(2) : 281–312. https://doi.org/10.1007/s11575-017-0336-9

131. Tastevin, Y. P., & Pliez, O. (2015) La discrète filière de l'autorickshaw: Une ethnographie de la mondialisation. *Revue Française de Socio-Economie*, Hors-série(2) : 121–137. https://doi.org/10.3917/rfse.hs1.0121

132. Terjesen, S., & Elam, A. (2009) Transnational entrepreneurs' venture internationalization strategies: A practice theory approach. *Entrepreneurship Theory and Practice*, 33(5): 1093–1120. https://doi.org/10.1111/j.1540-6520.2009.00336.x

133. Urbano, D., Toledano, N., & Ribeiro-Soriano, D. (2011) Socio-cultural factors and transnational entrepreneurship: A multiple case study in Spain. *International Small Business Journal,* 29(2): 119–134. https://doi.org/10.1177/0266242610391934

134. Valenzuela-Garcia, H., Güell, B., Parella, S., Molina, J. L., & Lubbers, M. J. (2018) Placing migrant entrepreneurship: Migrant economy debates through new spatial lenses. *Sociologica*, 12(2): 39–56. https://doi.org/10.6092/issn.1971-8853/8619

135. Valenzuela-Garcia, H., Parella, S., & Güell, B. (2017) Revisiting the 'ethnic enclave economy': Resilient adaptation of small businesses in times of crisis in Spain. *International Journal of Anthropology and Ethnology*, 1(1). https://doi.org/10.1186/s41257-017-0008-5

136. Van Dongen, E. (2019) Localizing ethnic entrepreneurship: "Chinese" chips shops in Belgium, "traditional" food culture, and transnational migration in Europe. *Ethnic and Racial Studies*, 42(15): 1–19. https://doi.org/10.1080/01419870.2018.1540788

137. Veréb, V. N., & Ferreira, J. J. (2018) Transnational entrepreneurship as a win-win scenario of international knowledge spillover. *Journal of the Knowledge Economy*, 9(2): 446–472. https://doi.org/10.1007/s13132-017-0496-7

138. Vershinina, N., Rodgers, P., Mcadam, M., & Clinton, E. (2019) Transnational migrant entrepreneurship, gender and family business. *Global Networks*, 19(2): 238–260. https://doi.org/10.1111/glob.12225

139. Villares-Varela, M. (2018) Negotiating class, femininity and career: Latin American migrant women entrepreneurs in Spain. *International Migration*, 56(4) : 109–124. https://doi.org/10.1111/imig.12361

140. Villares-Varela, M., & Essers, C. (2019) Women in the migrant economy. A positional approach to contextualize gendered transnational trajectories. *Entrepreneurship & Regional Development*, 31(3–4): 213–225. https://doi.org/10.1080/08985626.2018.1551789

141. Wahlbeck, Ö. (2013) Mixed embeddedness and the dynamics of self-employment among Turkish immigrants in Finland. *Polish Sociological Review*, 184: 487–503.

142. Wahlbeck, Ö. (2018a) The transferability and mobilisability of transnational resources: The case of Turkish entrepreneurs in Finland. *Nordic Journal of Migration Research*, 8(4): 237–244. https://doi.org/10.2478/njmr-2018-0027

143. Wahlbeck, Ö. (2018b) Combining mixed embeddedness and transnationalism: The utilization of social resources among Turkish migrant entrepreneurs. *Sociologica*, 12(2): 73–86. https://doi.org/10.6092/issn.1971-8853/8623

144. Walton-Roberts, M. (2011) Immigration, trade and 'ethnic surplus value': A critique of Indo-Canadian transnational networks. *Global Networks*, 11(2): 203–221. https://doi.org/10.1111/j.1471-0374.2011.00318.x

145. Wang, Q., & Liu, C. Y. (2015) Transnational activities of immigrant-owned firms and their performances in the USA. *Small Business Economics*, 44(2): 345–359. https://doi.org/10.1007/s11187-014-9595-z

146. Webster, N. A. (2017) Rural-to-rural translocal practices: Thai women entrepreneurs in the Swedish countryside. *Journal of Rural Studies*, 56: 219–228. https://doi.org/10.1016/j.jrurstud.2017.09.016

147. Wong, L., & Primecz, H. (2011) Chinese migrant entrepreneurs in Budapest: Changing entrepreneurial effects and forms. *Journal of Asia Business Studies*, 5(1): 61–76. https://doi.org/10.1108/15587891111100804

148. Yeung, H. W. (2009) Transnationalizing entrepreneurship: A critical agenda for economic geography. *Progress in Human Geography*, 33(2): 210–235. https://doi.org/10.1177/0309132508096032

149. Yousafzai, S., Fayolle, A., Saeed, S., Henry, C., & Lindgreen, A. (2019) The contextual embeddedness of women's entrepreneurship: Towards a more informed research agenda. *Entrepreneurship & Regional Development*, 31(3–4): 167–177. https://doi.org/10.1080/08985626.2018.1551786

150. Zack, T. (2015) 'Jeppe' Where Low-End Globalisation, Ethnic Entrepreneurialism and the Arrival City Meet. *Urban Forum*, 26(2): 131–150. https://doi.org/10.1007/s12132-014-9245-1

151. Zani, B. (2019) Gendered transnational ties and multipolar economies: Chinese migrant women's WeChat commerce in Taiwan. *International Migration*, 57(4): 232–246. https://doi.org/10.1111/imig.12526

152. Zapata-Barrero, R., & Hellgren, Z. (2019) Harnessing the potential of Moroccans living abroad through diaspora policies? Assessing the factors of success and failure of a new structure of opportunities for transnational entrepreneurs. *Journal of Ethnic and Migration Studies*. https://doi.org/10.1080/1369183X.2018.1559997

153. Zapata-Barrero, R., & Rezaei, S. (2019) Diaspora governance and transnational entrepreneurship: The rise of an emerging social global pattern in migration studies. *Journal of Ethnic and Migration Studies*. https://doi.org/10.1080/1369183X.2018.1559990

154. Zhou, Y., & Hsu, J.-Y. (2011) Divergent engagements: Roles and strategies of Taiwanese and mainland Chinese returnee entrepreneurs in the IT industry. *Global Networks*, 11(3): 398–419. https://doi.org/10.1111/j.1471-0374.2010.00302.x

155. Zolin, R., & Schlosser, F. (2013) Characteristics of immigrant entrepreneurs and their involvement in international new ventures. *Thunderbird International Business Review*, 55(3): 271–284. https://doi.org/10.1002/tie.21543
